# Aplicativos móveis direcionados aos idosos para autogerenciamento do cuidado: revisao de escopo*

**DOI:** 10.15649/cuidarte.2594

**Published:** 2023-05-28

**Authors:** Manoelise Linhares Ferreira-Gomes, Cristina da Silva-Fernandes, Maria Gabrieli Aguiar-de-Sousa, Raimunda Leandra Bráz-da-Silva, Illeanne de Jesus Manhiga-da-Costa-Silva, Lívia Moreira-Barros

**Affiliations:** 1 . Universidade Estadual do Ceará (UECE), Fortaleza-CE, Brasil. Email: manoeliselfg@gmail.com Universidade Estadual do Ceará Universidade Estadual do Ceará Fortaleza CE Brazil manoeliselfg@gmail.com; 2 . Universidade Federal do Ceará (UFC), Fortaleza-CE, Brasil. Email: cristina.sednanref@gmail.com Universidade Federal do Ceará Universidade Federal do Ceará Fortaleza CE Brazil cristina.sednanref@gmail.com; 3 Universidade Estadual Vale do Acaraú (UVA), Sobral- CE, Brasil. Email: gabrielleaguiargg@gmail.com Universidade Estadual do Vale do Acaraú Universidade Estadual Vale do Acaraú Sobral CE Brazil gabrielleaguiargg@gmail.com; 4 . Universidade Estadual Vale do Acaraú (UVA), Sobral- CE, Brasil. Email: leandrabraz7@gmail.com Universidade Estadual do Vale do Acaraú Universidade Estadual Vale do Acaraú Sobral CE Brazil leandrabraz7@gmail.com; 5 . Universidade da Integragáo Internacional da Lusofonia Afro-Brasileira (UNILAB), Redengáo-CE, Brasil. Email: Ileannesilva3@gmail.com Universidade Federal de Integração Lusofonia Afro-Brasileira Universidade da Integragáo Internacional da Lusofonia Afro-Brasileira Redengáo CE Brazil Ileannesilva3@gmail.com; 6 . Universidade da Integragáo Internacional da Lusofonia Afro-Brasileira (UNILAB), Redengáo-CE, Brasil. Email: livia.moreirab@hotmail.com Universidade da Integração Internacional da Lusofonia Afro-Brasileira Universidade da Integragáo Internacional da Lusofonia Afro-Brasileira Redengáo CE Brazil livia.moreirab@hotmail.com

**Keywords:** Enfermagem, Saúde do Idoso, Aplicativos Móveis, Autogestao, Nursing, Health of the Elderly, Mobile Applications, Self-Management, Enfermería, Salud del Anciano, Aplicaciones Móviles, Automanejo

## Abstract

**Introdujo:**

o uso de aplicativos móveis pode facilitar o autogerenciamento em saúde e oportunizar a autonomia dos idosos no seu autocuidado. Objetivo: mapear a produgáo científica sobre aplicativos móveis para autogerenciamento do cuidado direcionados aos idosos.

**Materiais e Métodos:**

revisao de escopo realizada no período de setembro de 2020 a janeiro de 2021, a partir das bases de dados: MEDLINE, SciELO, Scopus, Web of Science e Science Direct, mediante a estratégia de busca: (“Self management” OR “Self-care”) AND (Elderly OR “Old man”) AND (“Mobile Applications” OR Smartphone OR “Cell phone”), com a inclusao de artigos que tratassem do uso de aplicativos móveis por idosos para o autogerenciamento do cuidado, sem delimitado de tempo e idioma.

**Resultados:**

a amostra final compós-se de 14 artigos, categorizados em tres vertentes de gerenciamento, a saber: medicamentos, comorbidades e práticas saudáveis. Na maioria dos estudos, os aplicativos foram direcionados ao autogerenciamento dos medicamentos, seguidos dos cuidados de condigóes crónicas e por último a autoavaliagáo do risco de quedas e tratamentos nao-farmacológicos da dor.

**Discussao:**

esta revisao contribui para a prática clínica e pesquisa em enfermagem, uma vez que seus resultados apontam o que há publicado sobre o desenvolvimento e uso de aplicativos móveis por idosos para o autogerenciamento do cuidado.

**Condusdes:**

o uso de aplicativos móveis facilita o autocuidado da populagao idosa, principalmente, na gestao de medicamentos para condigóes crónicas.

## Abstract (en)

**Introduction:**

the use of mobile applications can facilitate self-management in health and provide opportunities for the autonomy of the elderly in their self-care.

**Objective:**

to map the scientific production on mobile applications for self-management of care aimed at the elderly.

**Materials and Methods:**

scope review carried out from September 2020 to January 2021, based on the databases: MEDLINE, SciELO, Scopus, Web of Science and Science Direct , using the search strategy: (“Self-management ” OR “Self- care ”) AND ( Elderly OR “ Old man ”) AND (“Mobile Applications ” OR Smartphone OR “ Cell phone ”), with the inclusion of articles that dealt with the use of mobile applications by the elderly for self-management of care, without limitation of time and language. Results: the final sample consisted of 14 articles, categorized into three aspects of management, namely: medications, comorbidities, and healthy practices. In most studies, the applications were directed to self-management of medications, followed by care for chronic conditions and finally self-assessment of the risk of falls and non-pharmacological pain treatments.

**Discussion:**

this review contributes to clinical practice and research in nursing, since its results point to what has been published about the development and use of mobile applications by the elderly for self-management of care.

**Conclusions:**

the use of mobile applications facilitates self care in the elderly population, especially in the management of medications for chronic conditions.

## Resumen

**Introducción:**

el uso de aplicaciones móviles puede facilitar la autogestión en salud y brindar oportunidades para la autonomía de los ancianos en su autocuidado.

**Objetivo:**

mapear la producción científica sobre aplicaciones móviles para la autogestión del cuidado dirigido al anciano.

**Materiales y Métodos:**

revisión de alcance realizada desde septiembre de 2020 hasta enero de 2021, con base en las bases de datos: MEDLINE, SciELO, Scopus, Web of Science y Science Direct , utilizando la estrategia de búsqueda: (“Self-management” OR “Self-care”) AND (Elderly OR “Old man”) AND (“Mobile Applications” OR Smartphone OR “Cell phone”),, con la inclusión de artículos que trataron sobre el uso de aplicaciones móviles por parte de los adultos mayores para la autogestión del cuidado, sin limitación de tiempo e idioma.

**Resultados:**

la muestra final estuvo compuesta por 14 artículos, categorizados en tres aspectos del manejo, a saber: medicamentos, comorbilidades y prácticas saludables. En la mayoría de los estudios, las aplicaciones se dirigieron a la autogestión de medicamentos, seguida de la atención de las condiciones crónicas y, finalmente, la autoevaluación del riesgo de caídas y los tratamientos no farmacológicos del dolor.

**Discusión:**

esta revisión contribuye para la práctica clínica y la investigación en enfermería, ya que sus resultados apuntan a lo publicado sobre el desarrollo y uso de aplicaciones móviles por parte de los ancianos para la autogestión del cuidado.

**Conclusiones:**

el uso de aplicaciones móviles facilita el autocuidado en la población adulta mayor, especialmente en el manejo de medicamentos para condiciones crónicas.

## Introdujo

A transigáo demográfica tem contribuido para o crescimento do índice de envelhecimento como fenómeno mundial, caracterizado pelo processo de senescencia, permeado por mudanzas irreversíveis, e náo patológicas, com o surgimento de inabilidades, e o aumento da fragilidade e dependencia, caracterizado pela senilidade^1^. A Organizado Mundial da Saúde (OMS) classifica por idosos todos os sujeitos com idade superior a 65 anos nos países desenvolvidos; já nos em desenvolvimento, essa condigáo é antecipada para 60 anos^2^.

No mundo, em 2020, havia 1,1 bilháo de idosos, com projegáo de 3,1 bilhóes em 2100, o que converge com o cenário brasileiro, que apresentava 29,9 milhóes em 2020 e previsáo de 72,4 milhóes em 2 1 00^3^. Desse modo, é pertinente a preocupado dos profissionais da saúde, a exemplo da Enfermagem, acerca da seguranza e cuidado desse público.

Nesse sentido, tem-se percebido avanzos na criado de tecnologias de informado e comunicado (TIC) associadas a saúde, o que oportuniza melhorias na qualidade de vida dos idosos^4^. O uso de smartphones tem propiciado recursos para otimizar a promodo da autonomia e autogerenciamento em saúde. Nos dispositivos eletrónicos, as intervengóes sáo desenvolvidas mediante aplicativos na internet, executados via software, que permitem a interagáo entre os sujeitos^5^. Além disso, a utilizado desses recursos pode aperfeigoar os conhecimentos dos idosos e aprimorar o autocuidado^4^.

Estudo desenvolvido na Suécia identificou que os idosos tem optado pelo uso de aplicativos móveis, pois favorecem o relato dos problemas aos profissionais de saúde, sendo por eles monitorados^4^. Acredita-se que os aplicativos tentam oportunizar planos de autogerenciamento do cuidado^6^.

O autogerenciamento do cuidado é definido como processos e comportamentos para gerenciar a condigáo de saúde, a exemplo do uso de medicamentos, obtengáo de prescrigóes e mudangas no estilo de vida, e ocorre no contexto do indivíduo, família, comunidade e sistemas de saúde, e pode ser influenciado por fatores externos^7^.

Um estudo de revisáo sistemática inferiu que os aplicativos podem auxiliar no manejo da dor5 e na seguranga frente ao risco de quedas. Diante disso, o autogerenciamento em saúde oportuniza a autonomia e a capacidade de intervengáo dos idosos no seu autocuidado^8^.

Salienta-se a importancia da Educado em Saúde com enfoque no manuseio de aplicativos com temáticas relacionadas aos cuidados de Enfermagem. Faz-se necessário o incentivo a estudos acerca da utilizagáo de TIC como intervengáo de Enfermagem, a fim de contribuírem com a Prática Baseada em Evidencias^5^^,^^8^.

Justifica-se este estudo a partir da necessidade de mapear a produgáo científica sobre a utilizagáo de aplicativos, em dispositivos móveis, como intervengáo de enfermagem frente ao processo saúde- doenga de idosos. Verifica-se a relevancia da pesquisa na produgáo teórica sobre o autogerenciamento do cuidado por meio dos aplicativos de smartphones. Assim, o estudo objetiva mapear a produgáo científica sobre aplicativos móveis para autogerenciamento do cuidado direcionados aos idosos.

## Materiais e Método

Revisáo de escopo, realizada no período de setembro de 2020 a janeiro de 2021, a partir do referencial teórico-metodológico The Joanna Brigs Institute for Scoping Reviews^9^.

O desenho do estudo foi integrado aplicando-se a metodología Populagáo, Conceito e Contexto (PCC) para nortear a coleta de dados^9^. A estratégia PCC é uma mnemónica que auxilia na identificado dos tópicos-chave: Populado, Conceito e Contexto. A populado elencada foram os idosos, o Conceito englobou o autogerenciamento do cuidado e o Contexto está relacionado ao uso de aplicativos móveis em diferentes cenários. Conciliando os tópicos-chave da PCC com o objetivo do estudo, a constituiu-se a questáo de pesquisa: quais aplicativos móveis para autogerenciamento do cuidado, direcionados aos idosos, estáo descritos na literatura?

Realizou-se buscas nas seguintes bases de dados: National Library of Medicine-USA (Medline), Scientific Electronic Library Online (SciELO), Scopus, Web of Science e Science Direct, mediante descritores identificados no Medical Heading Subjects (MeSH) e Descritores em Ciencias da Saúde (DeCS), com os quais definiu-se a estratégia de busca: (“Self-management” OR “Self-care”) AND (Elderly OR “Old man”) AND (“Mobile Applications” OR Smartphone OR “Cell phone”).

Para coleta dos dados, dois revisores realizaram, de forma independente, a leitura de título e resumo das publicagóes para a selegáo dos estudos. Os critérios de inclusáo foram: artigos que responderam a questáo de pesquisa, sem delimitado de tempo e idioma. Excluiu-se as publicagóes duplicadas.

A extragáo dos dados ocorreu mediante a utilizagáo de instrumento semiestruturado desenvolvido pelos autores, que incluiu informagóes acerca do título, autores, ano de publicagáo, periódico, método, principais resultados, conclusáo e nível de evidencia, a saber: nível I - metanálises, estudos controlados e randomizados; nivel II - ensaio clínico randomizado (ECR); nivel III - estudos quase-experimentais; nivel IV - estudos descritivos, náo experimentais ou qualitativos; nível V - relatos de experiencia e de caso; e nível VI - opiniáo e consensos de especialistas^10^.

Os dados foram analisados a partir de recomendagóes da The Joanna Brigs Institute for Scoping Reviews9, categorizados em tres vertentes de gerenciamento, a saber: medicamentos, comorbidades e práticas saudáveis; agrupados em quadro descritivo.

## Resultados

**Figura 1:**
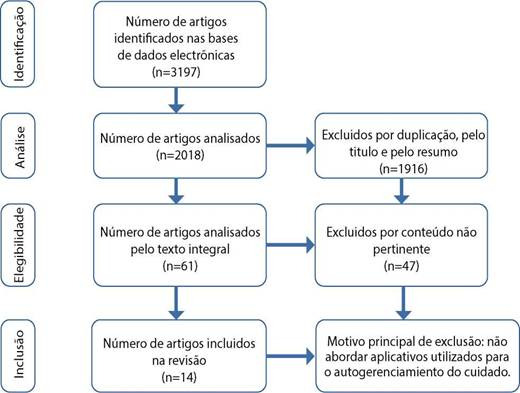
Artigos.

Foram incluídos 14 artigos. A selegáo dos estudos ocorreu conforme recomendado pelo Preferred Reporting Items for Systematic Reviews and Meta-Analyses (PRISMA)(9), de acordo com a Figura 1.

Em relagáo as perguntas temáticas, optou-se por incluir questóes abertas, de múltipla escolha, verdadeiro ou falso, além de situagóes-problema.

Dos documentos selecionados, todos sáo de língua inglesa, dois tiveram publicagáo em 2014, um em 2015, 2016, 2017 e 2018, tres em 2019 e cinco em 2020. Os artigos foram publicados nos periódicos: JMIR Mhealth Uhealth, Journal of Medical Internet Research, Medicine, Int. J. Healthcare Technology and Management, Journal of Biomedical Informatics, JMIR aging, Heathcare Informatics Research, S. Karger, JMIR Res Protoc, BMC Health Services Research, Plos One, International Journal of Medical Informatics, Jpn J Nurs Sci.

Houve prevaléncia de estudos descritivos qualitativos (cinco), pesquisas quase-experimentais (quatro), Ensaio Clínico Randomizado (trés), método misto (um) e analítico (um). Quanto aos níveis de evidencias, trés foram classificados como nivel II, quatro como nivel III, e sete como nivel IV. Em relagáo ao país de desenvolvimento dos estudos, dois foram no Canadá, Japáo, Estados Unidos, Espanha e Alemanha; e um na Noruega, Inglaterra, Coreia do Sul e China.

O Quadro 1 apresenta a síntese dos estudos incluídos na revisáo, contendo objetivo, amostra, aplicativo utilizado, desenho do estudo e as principais conclusóes. Salienta-se que os artigos foram identificados pela letra “A” seguida da ordem de análise.

**Quadro 1:** Caracterizado dos estudos incluidos na revisao. Sobral- CE, Brasil, 2021.

	Objetivo	N	Aplicativos utilizados	Desenho do estudo	Principáis conclusoes
A3^13^	Analisar como um aplicativo de autogestao de medicamentos afeta a adesao terapéutica em pacientes idosos.	24	Plano de medicado	Estudo experimental do tipo antes e depois	O aplicativo móvel aumentou a adesao medicamentosa em usuários idosos submetidos ao seu uso.
A4^14^	Desenvolver e avaliar um aplicativo que transforma códigos de barras associados a medicamentos em instruyes verbais para pacientes idosos.	61	TUMEDICINA	Estudo quase- experimental	Os resultados do estudo apoiam o uso dessa tecnologia para aumentar a seguranza dos pacientes que fazem uso de múltiplos medicamentos.
A5^15^	Explorar como idosos com Diabetes Tipo 2 e Hipertensao usam um aplicativo desenvolvido para ajudar a gerenciar essas condioes.	12	TAPESTRY- CM Healthy Lifestyle	Estudo descritivo	Apesar da avaliaao positiva dos participantes, sugeriu-se melhorias em relaao ao conteúdo e layout do aplicativo.
A6^16^	Examinar o uso de monitores de saúde para mudanzas no estilo de vida entre idosos com Diabetes.	9	Lose It	Estudo analítico	Os resultados sao positivos, mas sugerem novos estudos com grupo controle para confirmar os resultados.
_A7_^17^	Desenvolver um aplicativo que auxilia o autogerenciamento de Diabetes Tipo 2 e Pré-diabetes.	522	GlucoNote	Ensaio Clínico Randomizado	O GlucoNote ofereceu oportunidade para autogerenciamento das condioes clínicas analisadas.
_A8_^18^	Avaliar a aceitabilidade dos usuários sobre um aplicativo para autogerenciamento de Diabetes.	26	Few Touch, One Touch	Estudo descritivo	Observou-se positividade para aceitabilidade prática e social, o que fomenta a utilizado da ferramenta em outros cenários.
A9^19^	Examinar a usabilidade, por pacientes diabéticos, de um glicosímetro conectado a smartphones.	12	iBG-Star	Estudo descritivo	Os autores apontam que para sucesso da tecnologia é necessário o desenvolvimento de um servido seguro que permita, aos pacientes, controlar a transferéncia de dados entre pacientes e profissionais.
A10^20^	Desenvolver um aplicativo móvel baseado no programa de apoio a autogestao para determinar sua eficácia e efeitos em pacientes idosos que realizam hemodiálise.	40	Sistema de feedback	Estudo descritivo	Os autores concluiram que outros dispositivos e programas devem ser elaborados para intensificar o envolvimento de profissionais da saúde na detecao dos risco em pacientes idosos que necessitam realizar hemodiálise.
A11^21^	Determinar a eficácia de uma série de aplicativos móveis para melhorar a gestao do autocuidado em saúde.	282	Programa proativo de saúde móvel	Ensaio clínico randomizado	Os aplicativos podem ajudar os idosos a administrarem melhor a saúde na comunidade, além de possibilitar o autocuidado.
A12^22^	Fornecer dados sobre o tratamento nao farmacológico da dor baseado em ferramentas da Web.	25	Pain e-Health Platform (PEP)	Estudo descritivo	O PEP pode otimizar o tratamento nao farmacológico, principalmente, da dor lombar crónica. Ademais, oportuniza o desenvolvimento de plataformas informativas acerca de outras dores e condioes crónicas.
A13^23^	Comparar o uso de um aplicativo móvel 3D, que permite pacientes idosos realizarem tarefas autocuidado, com um papel 2D equivalente.	34	Guide to measure-3D	Estudo de método misto	Este estudo revelou que idosos usando o Guide Measure-3D alcanaram melhores níveis de satisfago e confianza em comparado ao papel 2D. Dessa forma, os resultados sao significativos e promissores para superar o abandono de equipamentos em papel.
A14^24^	Desenvolver e determinar a viabilidade clínica de um aplicativo de autocuidado para pacientes com Gota.	56	GoutCare	Estudo quase- experimental	O aplicativo contribuiu para melhorar o desempenho do autocuidado e a qualidade de vida dos pacientes com Gota. Ademais, o GoutCare oportunizou aos participantes o conhecimento sobre Gota, atitudes de autogestao, percepao social e autogerenciamento da doena.

### Gerenciamento de medicamentos

Os artigos abordaram como principal funcionalidade dos aplicativos o gerenciamento de medicamentos^11^^-^^14^. Foram analisadas as percepgóes de idosos acerca da usabilidade de quatro programas de gerenciamento de fármacos, mediante lembretes com informes sobre os medicamentos e suas interagóes^11^ e idosos em uso de polifarmácia utilizaram aplicativo, a fim de otimizar a adesao a terapia medicamentosa e viabilizar a seguranga desta^12^. Foi notabilizada a importancia do uso de aplicativo como intervengao eficaz no cuidado a idosos cardiopatas^13^. Além disso, um dos estudos apresentou tecnologia potencializadora da seguranga dos partícipes em tratamento medicamentoso^14^.

### Gerenciamento de comorbidades

Os estudos apresentaram variados desfechos acerca do gerenciamento de comorbidades^15^^-^^20^^,^^24^. Um dos estudos discutiu sobre o estilo de vida dos idosos com Diabetes Mellitus (DM) e Hipertensao Arterial Sistemica (HAS)^15^, enquanto outro analisou a usabilidade de dois aplicativos relativos ao DM, com enfoque no monitoramento de dieta e da pressao arterial (PA)^16^.

Ainda sobre doengas crónicas, os artigos trataram do autogerenciamento do cuidado frente ao DM, bem como o monitoramento deste por glicosímetro conectado a um smartphone^17^^-^^19^.

Somou-se a isso o artigo que apresentou intervengao com pacientes em diálise, para otimizar seus conhecimentos e atitudes frente ao tratamento20. Outrossim, outro estudo tratou do manuseio e viabilidade clínica de aplicativo indicado para o autocuidado de sujeitos com Gota^24^.

### Gerenciamento de práticas saudáveis

Acrescentaram-se a esses os estudos sobre gerenciamento de práticas saudáveis^21^^-^^23^. Um dos artigos expós a relevancia do uso de aplicativo móvel entre idosos que vivem em comunidade, auxiliando- os em seus aspectos físico e psicossocial, o que oportuniza o autocuidado^21^. examinou a efetividade de ferramenta por meio de informagóes acerca do tratamento nao farmacológico da dor^22^, e exibiu programa que permitiu a realizagao de atividades para autoavaliagao do risco de quedas, pelos beneficiários do servigo, no ambiente doméstico^23^.

## Discussao

Dentre os artigos analisados, quatro abordaram o autogerenciamento de medicamentos. Entende- se por essa prática o empoderamento dos sujeitos acerca da sua condigao de saúde e terapeutica medicamentosa, efetivando-a mediante autonomia e independencia. Um dos artigos demonstrou que mesmo sem experiencia no uso de smartphones, os idosos conseguiram usar o aplicativo^12^, o que corrobora com uma revisao sistemática realizada em 2019, que destacou o uso de aplicativos como propiciadores da adesao aos medicamentos, sendo mais eficientes do que as estratégias convencionais definidas pelo sistema de saúde^25^.

Estudo americano publicado em 2020 analisou a qualidade de vida de pacientes com Insuficiencia Cardíaca após o uso de aplicativos, e apontou mudangas clínicas satisfatórias na manutengao do autocuidado e adesao ao tratamento farmacológico^26^. Assim, faz-se necessária a elaboragao e validagao de aplicativos como estratégias norteadoras do autogerenciamento do cuidado, sendo essencial a avaliagao por profissionais que tenham expertise na temática de tecnologias digitais.

Entende-se por comorbidade a ocorréncia simultanea de dois ou mais agravos a saúde^27^, a exemplo das condigóes crónicas, as quais influenciam na funcionalidade dos idosos. Dessa forma, o autogerenciamento dessas enfermidades oportuniza o entendimento acerca das agóes promotoras de saúde e a participagao ativa no cuidado. Neste contexto, um dos estudos inferiu que o uso de mHealth, por sujeitos diabéticos com 60 anos ou mais, favoreceu mudangas no estilo de vida, tais como autorregulagao e desenvolvimento de habilidades na resolugao de problemas^16^, corroborando com uma revisao narrativa publicada em 2020, que apontou o estímulo de hábitos saudáveis mediante aplicativos móveis^28^.

Todavia, outro artigo apontou que o uso de aplicativos nao interferiu de forma significativa na motivagao e adesao ao comportamento das pessoas avaliadas^20^, o que reforga os achados do estudo^29^ que afirmaram dificuldades no manuseio de aplicativos de smartphones para o autocuidado, prejudicando a implementagao de práticas que norteiam a melhoria da qualidade de vida.

Por ser a categoria profissional que atua no gerenciamento do cuidado, a Enfermagem se destaca como ciéncia essencial na promogao do conforto e das habilidades sociais envoltas nas atividades básicas de vida diária (AVDs), viabilizadas mediante aplicativos móveis, o que exige dos profissionais coragem, raciocínio clínico e paciéncia na elaboragao de agóes educativas. Além disso, os enfermeiros, principalmente, os que atuam na atengao primária a saúde sao essenciais no processo de cuidado dos idosos, bem como no desenvolvimento e aplicagao de tecnologias que auxiliam no seu autocuidado e consequente autogerenciamento de suas condigóes de saúde, o que corrobora com a autonomia e independéncia desse público.

Destaca-se como limitagao desta revisao o nível de evidéncia dos estudos incluídos, pois a maioria se tratava de pesquisas descritivas qualitativas. Apontam-se como contribuigóes para a prática clínica e pesquisa na área de enfermagem, o fato de os resultados desta revisao destacarem o desenvolvimento e uso de aplicativos móveis por idosos para o autogerenciamento do cuidado, em especial, a autogestao de medicagóes e o controle da dieta. Logo, os achados deste estudo poderao sustentar a elaboragao de outras ferramentas tecnológicas que auxiliem no autogerenciamento de cuidados dos idosos, bem como outros públicos.

## Conclusoes

O presente estudo expós aplicativos móveis relacionados a autogestao de fármacos no domicílio, bem como apresentou estratégias de autogerenciamento em situagóes clínicas como DM, Gota, tratamentos dialíticos e dor. Ademais, os aplicativos oportunizaram a autoavaliagao do risco de quedas.

Salienta-se a necessidade de estudos sinérgicos que abordem os aplicativos móveis como intervengao de Enfermagem junto a populagao idosa no contexto da assisténcia a saúde nos trés níveis de atengao.
